# Sika deer antler as a novel model to investigate dental implant healing: A pilot experimental study

**DOI:** 10.1371/journal.pone.0200957

**Published:** 2018-07-31

**Authors:** Yun He, Dominik Fischer, Istabrak Hasan, Werner Götz, Ludger Keilig, Luisa Ziegler, Markus Abboud, Christoph Bourauel, Gerhard Wahl

**Affiliations:** 1 Endowed Chair of Oral Technology, University of Bonn, Bonn, Germany; 2 Department of Oral Surgery, Hospital of Stomatology, Southwest Medical University, Luzhou, China; 3 Raptor Centre and Wildlife Park Hellenthal, Hellenthal, Germany; 4 Clinic for Birds, Reptiles, Amphibians and Fish, Veterinary Faculty, Justus Liebig University Giessen, Giessen, Germany; 5 Department of Prosthetic Dentistry, Preclinical Education and Materials Science, University of Bonn, Bonn, Germany; 6 Department of Orthodontics, Oral Biology Laboratory, University of Bonn, Bonn, Germany; 7 Department of Prosthodontics and Digital Technology, School of Dental Medicine, Stony Brook University, Stony Brook, New York, United States of America; 8 Department of Oral Surgery, University of Bonn, Bonn, Germany; University of Otago, NEW ZEALAND

## Abstract

Dental implants are important tools for restoring the loss of teeth. The rapid growth and periodic regeneration of antlers make Sika deer a good and less invasive alternative model for studying bone remodelling in mammals. We developed a special loading device for antlers and analysed the bone reaction around unloaded implants and under immediate loading conditions until osseointegration occurred. In micro-computed tomography images, the density of antler tissue around the implants increased as the loading time increased. This finding was histologically confirmed by the good osseointegration observed in unloaded and loaded specimens. Antler tissue displays a similar healing process to human bone. The use of an antler model is a promising alternative for implant studies that does not require animal sacrifice.

## Introduction

Dental implants are important tools for restoring the loss of single teeth and fixing tooth implant-supported fixed partial dentures after accidents, disease or age-related loss of teeth [1]. Therefore, dental implants improve the quality of a patient’s life by improving aesthetics and phonetics and by decreasing bone resorption processes in the alveolar ridge. In particular, immediately loaded dental implants offer fast dental restoration and pain relief [1,2]. Research regarding complex bone remodelling processes occurring around dental implants is crucial to improve the function and acceptance of dental implants. Therefore, *in vivo* models are required, and animal trials have previously been conducted in pigs and dogs that were ultimately sacrificed [3,4]. This limitation restricted an intensive investigation of bone tissue due to ethical reasons.

Deer antlers represent well-exposed and rapidly growing bones that change and regenerate periodically. Thus, antlers may be used as a good model for studying bone remodelling in mammals [5–10]. Antlers start to grow from bony pedicles located on the head of male Sika deer in winter or spring and are enveloped in vascularised velvet, the periosteum, during growth [11]. The growth rate may reach up to 1.2 cm per day during the 70 days of the fastest growth period [11]. In summer, growth ceases, and the antlers are completely mineralised; in addition, the velvet is shed, thereby exposing the bare bone of the so-called hard antlers from July to August [11,12]. The development of an abscission layer across the base induces casting of the antlers to complete the cycle and to enable subsequent regrowth [13–16].

In our study, we used Sika deer antlers as a less invasive model to analyse the bone remodelling processes occurring around dental implants in different periods until complete osseointegration of the implants was achieved, and the antlers were shed. The use of a self-developed autonomous loading device [17] enabled an *in vivo* analysis under an immediate loading situation without sacrificing the animals or altering the normal behaviour of the deer. Micro-computed tomography (μCT) and histological analyses were used to reveal changes in bone structure and density during healing and to understand the general ossification process and the specific reaction of bone around implants. Finite element analyses were used to observe the biomechanical properties of the implant and the surrounding antler tissue.

## Materials and methods

### Animal selection

Six healthy 4-year-old male captive bred and tamed Sika deer (Cervus nippon) were utilised in this study. The median weight of the animals was 64 kg (all 50–70 kg). All animals were handled according to the policies and principles established by the German Animal Welfare Act and approved by the North Rhine-Westphalia State Agency for Nature, Environment and Consumer Protection as the competent authority (Permission No.: LANUV NRW, 84–02.04.2014.A462).

### Animal preparation and the surgical procedure

The animals were intramuscularly anaesthetised with 1.2–1.5 ml of Hellabrunn Mixture (100 mg of Ketamine and 125 mg of Xylazine per ml) [18] via distance immobilisation using a carbon dioxide injection gun (DAN-INJECT JM Standard injection rifle, DAN-INJECT Smith GmbH, Walsrode, Germany) [19]. After placing the deer on a surgical table in right lateral recumbency, anaesthesia was monitored by a visual inspection of breathing, auscultation of the heart and lungs, continuous monitoring of the rectal temperature and pulse oximetry (LifeVet P, Eickemeyer Medizintechnik für Tierärzte KG, Tuttlingen, Germany). A continuous oxygen supply was ensured by the use of a nasal tube and a flow rate between 1 and 5 l/min, depending on the breathing and oxygen parameters. A continuous intravenous drip infusion was applied via the lateral saphenous vein. The non-steroidal, anti-inflammatory agent meloxicam (Meloxidyl 20 mg/ml ad us vet., Ceva Tiergesundheit, Düsseldorf, Germany) was injected intramuscularly. In addition, local analgesia was applied by injecting 3–5 ml of lidocaine beneath branches of the zygomatic nerve at the antler’s base (Lidocaine 2%, B. Braun Melsungen, Melsungen, Germany, [20, 21].

**Fig 1:**
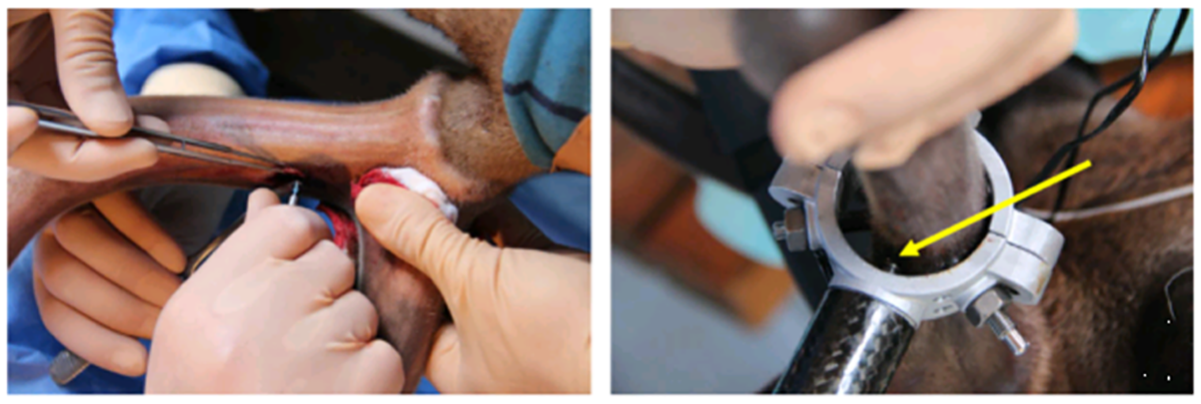
Dental implant inserted into the Sika deer antler and loaded. Insertion of a dental implant into the Sika deer antler (left). Each animal (deer no. 1–5) received two implants at a position near the branching point of the antler. One of the implants was loaded by an autonomous loading device controlled by a micro-computer. The implant was loaded by a motor driven thrust die (yellow arrow).

Before surgically raising the velvet from two 1.5 × 1.5-cm^2^-sized areas close to branches of the antler, disinfection was repeated with 70% alcohol and a 1% iodine tincture (Applichen GmbH, Darmstadt, Germany), and a longitudinal incision of 5 cm was then performed without releasing cuts to raise the velvet of both sides up to a 1-cm distance to the longitudinal incision. The implant sites were prepared by sequential drilling with a Ø 2.2-mm and a Ø 2.8-mm cylindrical twist drill and 800 U/min under sterile saline irrigation according to the manufacturer’s protocol. In five animals (deer no. 1–5), two Straumann-Standard Plus Roxolid^®^ soft tissue level implants (Institut Straumann AG, Basel, Switzerland) with a length of 10 mm and a diameter of 3.3 mm were inserted without thread-cutting in the left antler of each deer. The distance between the two implants was 2.5 cm. Roxolid^®^ is a titanium-zirconium alloy composed of approximately 15% zirconium and approximately 85% titanium and has been specifically designed for use in dental implantology [22]. The first implant received a ball abutment for transvelvet healing, and the second received a closure screw. The tissues collected from the antlers of the sixth animal (deer no. 6, control animal that did not receive an implant) using a trepan drill were used as a control. The velvet was finally sutured using Serafit 4/0 (SERAG-WIESSNER GmbH & Co. KG, Naila, Germany) and was subsequently covered with a Hansaplast spray bandage (Beiersdorf AG, Hamburg, Germany). The first implant was immediately vertically loaded via the custom-made loading device fixed with bone screws [17], whereas the second implant remained unloaded (Fig 1). The self-developed load control unit was attached to the right antler. No loading device was placed in the control animal.

### Follow-up and sample removal from the deer

Post-operatively, the animals and wounds were inspected daily for the absence of wound healing complications, alterations in general health, abnormal behaviours and reduced food intake. The correct placement of the loading devices was monitored daily. Animals were assigned randomly to different time points for implant removal, ranging from 3 to 6 weeks after surgery (deer no. 2: 3 weeks post-operation; deer no. 3: 4 weeks post-operation; deer no. 4: 5 weeks post-operation; and deer no. 5: 6 weeks post-operation). For this procedure, the animals were anaesthetised as described above, and vertically loaded implants and the surrounding tissues were collected using the trepan drill. The wound cavities were rinsed thoroughly and filled with a collagen sponge for local haemostasis (KOLLAGEN resorb, RESORBA Medical GmbH, Nuremberg, Germany). Finally, the velvet was sutured, and the Hansaplast spray bandage was applied. Loading devices and the load control unit were removed. The unloaded implants were kept in place and collected after antler shedding in February of the next year to investigate the osseointegration of the implants in a fully grown antler and the structure of antler tissue in comparison to human bone. Collected samples were immediately fixed in buffered formalin (4%) and subjected to μCT scanning and subsequent analyses.

### Antler density analysis

Specimens collected from antlers 3, 4, 5 and 6 weeks after implant insertion were used in this study to analyse changes in antler density at different healing periods. The specimens were scanned in a micro-computed tomography (μCT) scanner (SkyScan 1174, SkyScan Bruker-microCT, Kontich, Belgium) at 50 kV, 800 μA, and a rotation step of 0.25°. A 1-mm-thick aluminium filter was used for beam-hardening reduction. The exposure time was set to 2 s. The scan times were approximately 10 hours per sample. After scanning each sample, two calibration phantom rods (SkyScan, Kontich, Belgium) with bone mineral density (BMD) values of 0.25 and 0.75 g/cm^3^ calcium hydroxyapatite were scanned on the same day using the same parameters. With the scans and reconstructions of the samples and the calibration phantoms complete, the BMD calibration program was performed using a Bruker-MicroCT CT-Analyser (SkyScan, Kontich, Belgium). First, the attenuation coefficient (AC) value of the two phantoms was measured and entered in the preferences histogram calibration table. The BMD-AC formula was updated, and the calibration was completed. Second, 3 mm of peri-implant antler tissue was selected, and the BMD was automatically calculated and recorded as the mean ± standard deviation (SD).

### Histological analysis

Antler samples with integrated implants were fixed in formaldehyde, dehydrated and infiltrated with ultraviolet light-activated polymethylmethacrylate (PMMA, Technovit 72100^®^, Heraeus Kulzer, Hanau, Germany) for one week. This procedure was followed by a 3-day immersion in a 1:1 combination of PMMA and 2-hydroxyethyl methacrylate (GMA, Heraeus Kulzer). After subsequent immersion in 100% embedding medium, the samples were photopolymerised under high-power UV light sources in three steps. Later, parallel 100- to 200-μm-thick sections were cut from the specimens using a microsaw machine (EXAKT Advanced Technologies, Norderstedt, Germany), and sections up to 20 μm in thickness were polished down using a micro grinding system (EXAKT). Sections obtained from the grinding system were stained using toluidine blue without removing the plastic medium and were evaluated under a Zeiss-Axio-Imager^®^ light microscope (Zeiss, Jena, Germany) at original magnifications ranging between 5 and 50x.

### Numerical analysis

**Fig 2:**
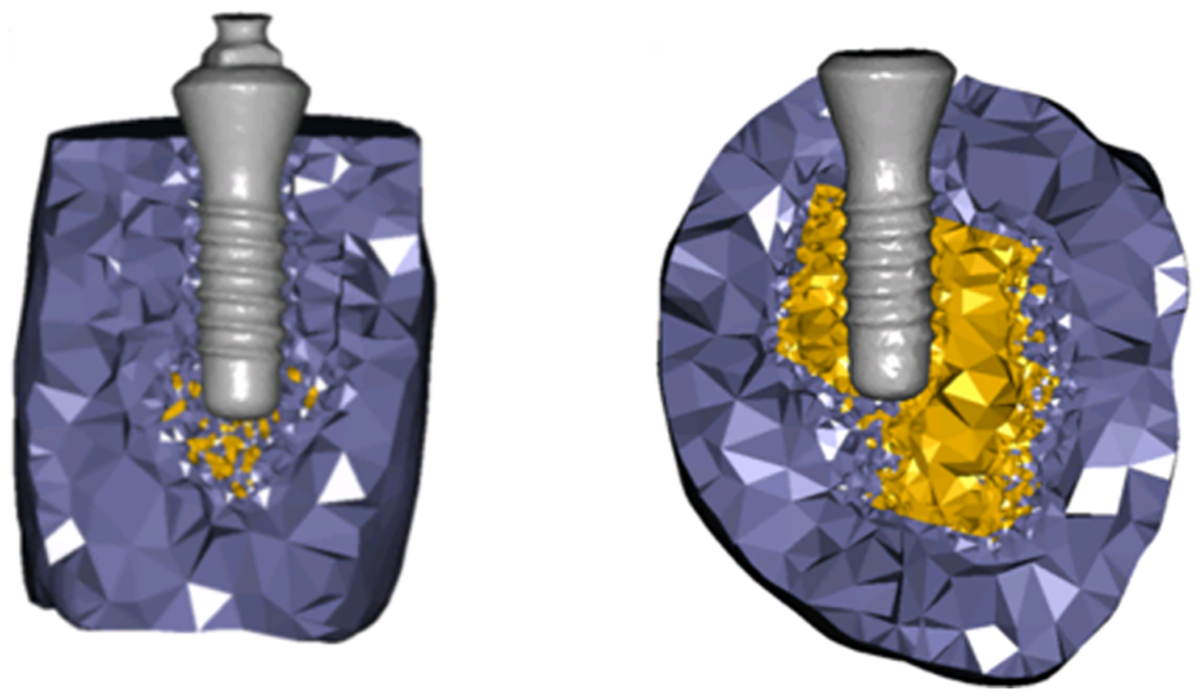
Example of finite element models of the specimens with and without loading. FE models created 6 weeks after loading (left); and an unloaded specimen from the same antler (right).

The μCT data from the four loaded and three unloaded specimens were imported into Mimics research 19.0 and 3-Matic research 9.0 (Materialise NV, Leuven, Belgium) for 3D reconstruction. Thereafter, the models were converted into 3D finite element (FE) models using 4-noded tetrahedral elements. The final models consisted of 235,297–258,158 elements for the specimens retrieved 3, 4, 5 and 6 weeks after loading and 532,709–1,116,651 elements for the unloaded models (Fig 2). The FE analysis was performed using the software package MSC.Marc/Mentat 2010 (MSC. Software, Santa Ana, CA, USA). All materials were assumed to be homogenous, isotropic and linearly elastic. Young’s modulus of the antler tissue was calculated from the density using the following formula [23]:

$$\text{E}=\{\begin{array}{ll} 2014\rho^{2.5} & \text{if}\;\rho \leq 1.25\;\text{g}/{\text{cm}}^{3}\\ 1763\rho^{3.2} & \text{if}\;\rho >1.25\;\text{g}/{\text{cm}}^{3} \end{array}$$

The material properties of the implant were calculated for Roxolid^®^ material (Young’s modulus: 98 GPa; Poisson’s ratio: 0.3; 22). A frictional contact with a frictional coefficient of 0.3 was defined between the implant and the antler tissue to simulate the immediate loading condition [24]. All models were constrained in all directions at the cutting surfaces and the bottom of the antler tissue. A linear vertical force of 10 N was applied on the implant. Stresses and strains were evaluated and compared for all of the models using the software package MSC.Marc/Mentat 2010.

## Results

### Animal health and behaviour after implantation and loading

Signs of infections in the antler, behavioural changes and signs of impairment in the deer were not observed during the trial. The antlers of all animals were shed regularly during the subsequent spring and were replaced with new and completely intact antlers within 2–3 months. Thus, the present trial did not disturb the normal behaviour of the deer or antler regeneration.

### Number of specimens included in the analysis

For the specimens that were loaded for 2 weeks post-operatively, the thickness of the antler tissue around the implant was less than 3 mm and was not suitable for histological analysis or BMD study. For this reason, four time points were selected for removing the samples after immediate loading, namely, 3, 4, 5 and 6 weeks.

Only three unloaded implant samples were collected after antler shedding due to the difficulty finding all the shed antlers in the Sika deer’s activity space. Therefore, three unloaded specimens were included in the analysis.

### Bone mineral density of antler tissue

The BMD of antler tissue around the implant increased significantly during the healing period under immediate loading conditions. The BMD values observed at 3, 4, 5 and 6 post-operative weeks were 0.31 ± 0.01 g/cm^3^, 0.92 ± 0.23 g/cm^3^, 1.54 ± 0.40 g/cm^3^ and 2.00 ± 0.53 g/cm^3^, respectively. The BMD values observed at 6 post-operative weeks were similar to those for healthy adult human cortical bone [24]. For unloaded specimens, the BMD values for antler tissue were lower than those for loaded implants. For example, in the deer segment obtained 6 weeks after implant insertion, the BMD values of antler tissue were 1.30 ± 0.11 g/cm^3^ around the unloaded implant and 2.00 ± 0.53 g/cm^3^ around the loaded implant.

## Histological results

### Unloaded specimens

**Fig 3:**
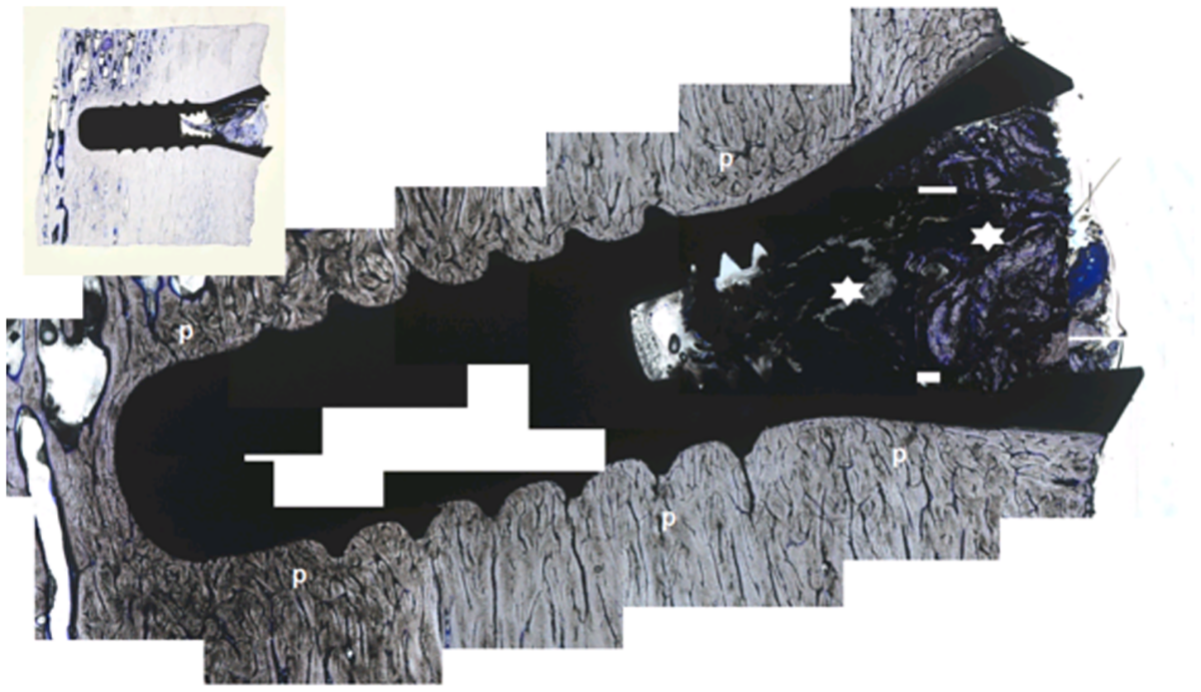
Unloaded implant in a Sika deer antler after 6 weeks from insertion. p = Peri-implant bone, asterisks = artefacts and detritus in the inside taper; toluidine blue staining, reconstruction and 5x magnification; Inset: overview of the inserted implant; 1:1.

**Fig 4:**
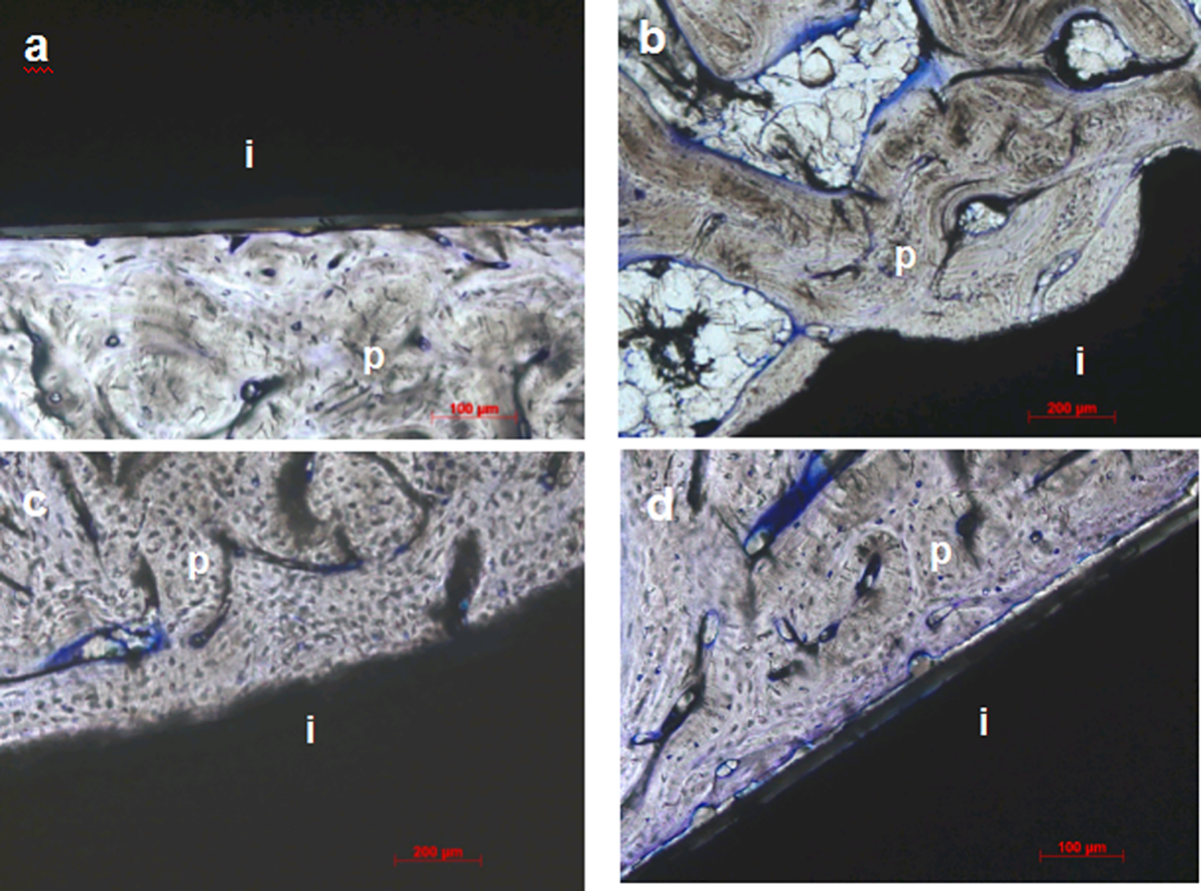
Detailed images of the interfaces from the unloaded implants in the Sika deer antler. a) High-magnification image of cortical peri-implant bone (midshaft), b) high-magnification image of periapical cancellous bone (images in both a and b were captured 3 weeks after insertion), c) high-magnification images of cortical peri-implant bone (crestal) and d) the peri-implant gap (crestal, images in both c and d were captured 6 weeks after insertion); toluidine blue staining, 10x magnification (b), 20x magnification (a, c and d); p = peri-implant bone, i = implant.

Specimens carrying unloaded implants collected after antler shedding displayed signs of excellent osseointegration (Figs 3 and 4). The peri-implant bone was mostly compact and peri-apically more cancellous with few fat marrow spaces. Growth was also visible in the crestal direction along the machined crestal implant surfaces (Fig 3). The crestal bony plateaus were smooth, contained only a thin layer of soft tissue remnants and showed no signs of bone resorption. A close bone contact forming a tight bone-implant interface was observed along the implant surface. At higher magnifications, e.g., 40 or 50x, single osteocytes were in very close proximity (maximal 5 μm) to the implant interface (Fig 4a–4c). Short and narrow peri-implant spaces of 10–20 μm were focally observed (Fig 4d). Substantial vascularisation was observed in perforating channels and osteons near (maximal 10 μm) and distal to the implant surface. Two defects were evaluated after explantation of the implants to investigate the healing outcome. In both defects, small apically located tears, a small amount of detritus and small bony fragments were observed. A soft tissue layer with dense connective tissue covered the defect bone surfaces.

### Loaded specimens

**Fig 5:**
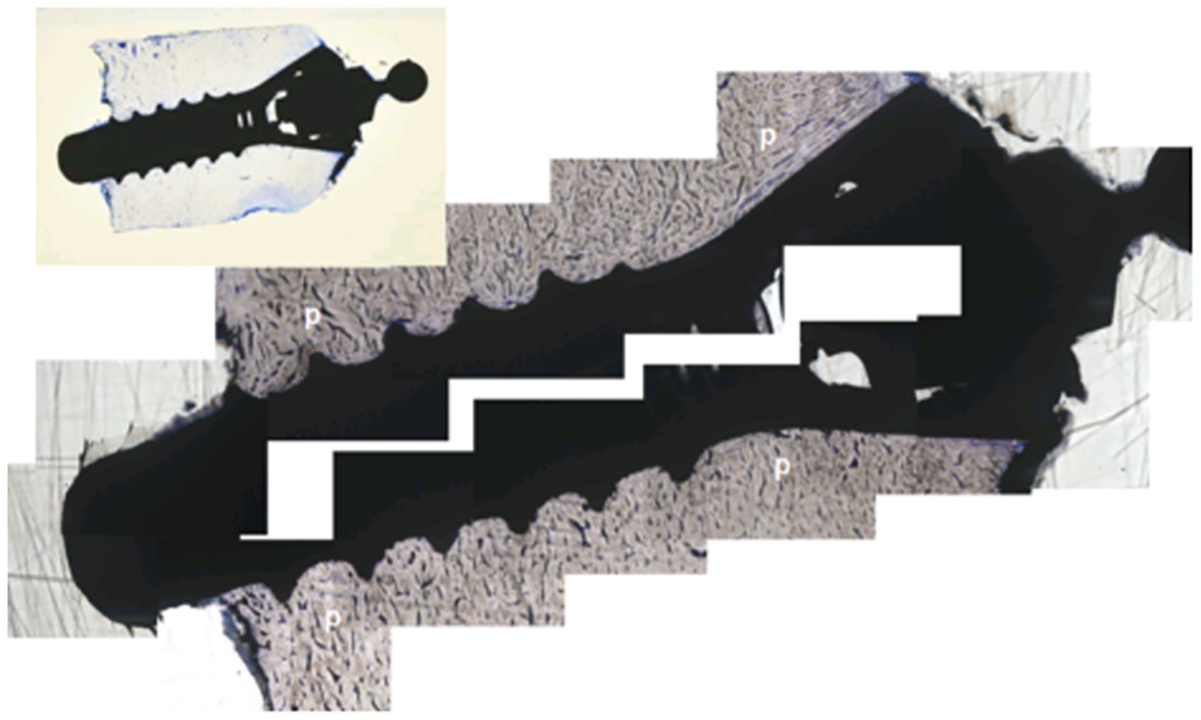
The loaded implant in the Sika deer antler 6 weeks after insertion. p = peri-implant bone; toluidine blue staining, reconstruction, and 5x magnification; Inset: overview of the inserted implant; 1:1.

**Fig 6:**
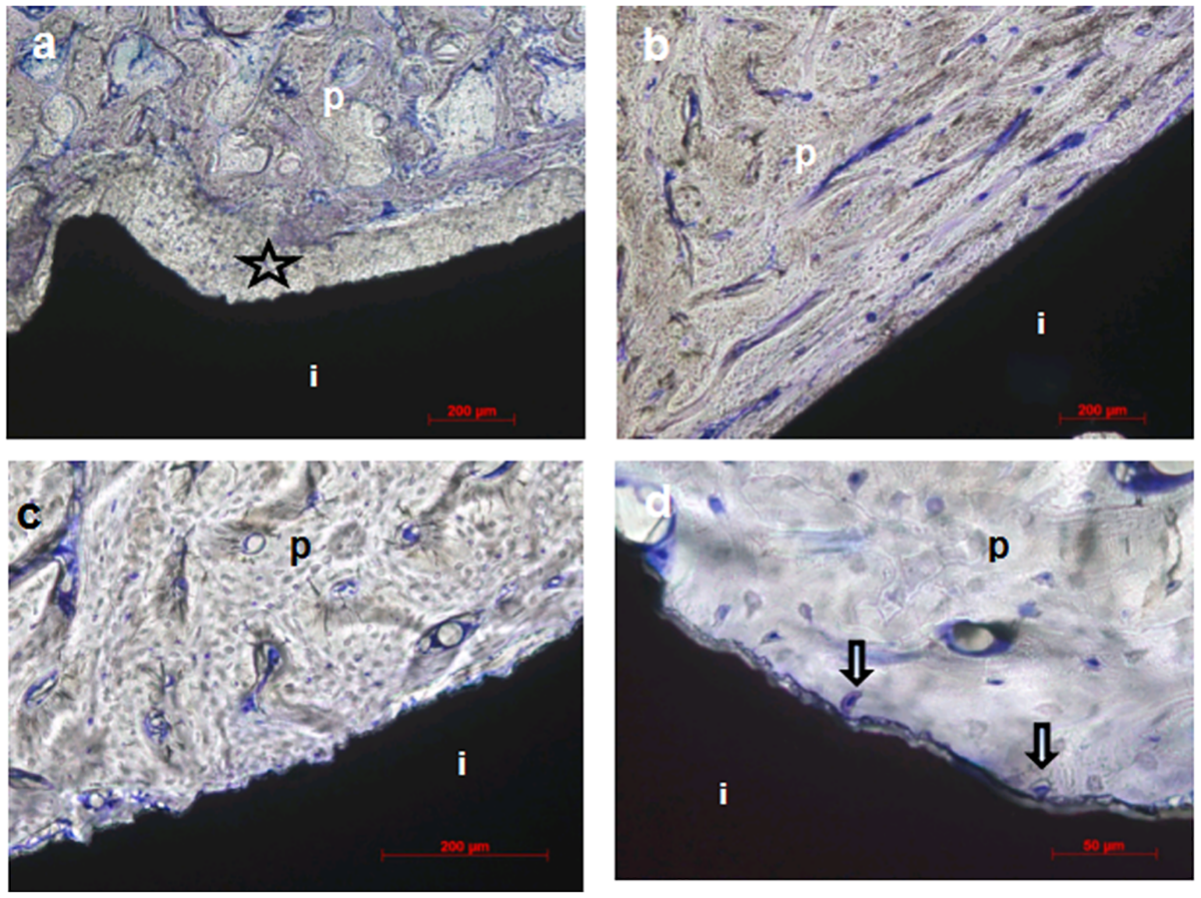
Detailed images of the interfaces from loaded implants in a Sika deer antler. a) Peri-implant gap along the implant body at 3 weeks after insertion, star: gap; b) high-magnification image of the compact bone along the midshaft implant surface at 6 weeks after insertion; c) high-magnification image of the compact bone as in b); please note the abundance of blue stained vessels in the peri-implant bone in b) and c). d) Osteocytes of the peri-implant bone are in close contact with the implant surface (open arrows) at 6 weeks after insertion; toluidine blue staining, 10x magnification (a and b), 20x magnification (c), 50x magnification (d); p = peri-implant bone, i = implant.

Osseointegration appeared to be insufficient in the specimens obtained at 3, 4 and 5 post-operative weeks. Peri-implant spaces with diameters up to 100 μm were observed along the implant bodies, ranging between half of the length and the entire length of the implant body. These spaces were filled with extravasations, blood cells, plaque and detritus. The peri-implant bone mostly consisted of a spongy or cancellous bone with good vascularisation. In the cervical and crestal regions of the implants, soft peri-implant tissue was present and likely originated from the pedicle skin or the periosteum of the antler. This soft tissue consisted of loose connective tissue covered by a multi-layered epithelium, focal round cell infiltration and granulation tissue. Osteogenesis was detected in the crestal region of the cancellous bone, particularly in specimens retrieved 3 and 4 weeks after surgery (deer nos. 2 and 3). However, similar to the unloaded implant specimens, the loaded implant specimen retrieved at 6 post-operative weeks exhibited very good osseointegration and a compact peri-implant bone with good vascularisation (Figs 5 and 6). Interestingly, the orientation of bone lamellation in the coronal areas was mainly longitudinal and parallel to the implant body axis (Fig 6).

## Numerical results

To evaluate the stress and strain, a 3-mm peri-implant antler tissue was selected. Stresses in antler tissue increased from 2.4 MPa (3 weeks after surgery) to 6.5 MPa (5 weeks after surgery) after immediate loading and decreased to 1.7 MPa after 6 weeks of loading. The values in the unloaded models displayed a similar range (1.0–1.3 MPa). The distribution of the stresses in antler tissues for all models was similarly concentrated in the antler tissue around the neck of the implant.

**Fig 7:**
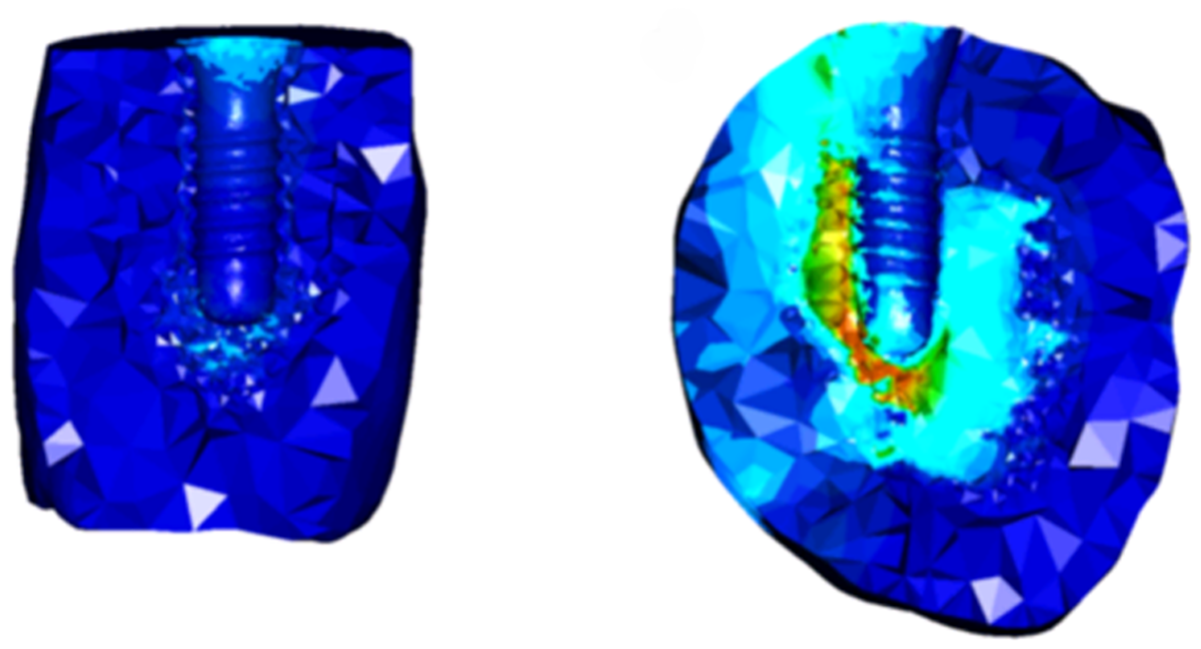
Example of strain in antler tissues in loading and unloaded models. Strains observed in the antler tissues 6 weeks after loading (left) and in the unloaded specimen from the same antler (right).

Strain in antler tissues decreased during the healing time for the loaded models, the 3-week (9,878 μstrain) and 6-week loaded models (49 μstrain), and illustrated the highest and lowest maximum strain values among the antler tissues. For the models from deer no. 2, the maximum strain in antler tissue in the loaded model was higher than that in the unloaded model. In contrast, the opposite results were obtained in the models from deer no. 5 (Fig 7).

## Discussion

Hard antlers are well suited mechanically since they are subject to high-impact loading and large bending moments [10,13,25,26]. The mechanical properties of hard antlers are highly anisotropic with the longitudinal elastic modulus, and higher bending, tensile and compressive strengths are observed in this direction than in the transverse direction [17]. Antlers dry out completely following the shedding of velvet [13] and transform into dead structures with the absence of further growth or bone remodelling until antler replacement [27].

Cortical porosity may vary along the main shaft [27]. The porosity is low in the lower and middle thirds of the shaft due to the more rapid completion of cortex formation prior to velvet shedding [27]. The more distal parts of the antler may be more porous, as velvet shedding interrupts the ongoing mineralisation process in the primary osteons. Thus, cortical porosity in the antler may not be the result of remodelling activity but may be the result of incomplete bone formation [16,27].

Antlers are predominantly composed of primary osteons [15,28]. In contrast, human bone is a secondary bone formed by the resorption of previously existing bone tissue and its replacement by new bone [26]. This process results in the formation of secondary osteons [29]. All secondary osteons include a prominent hyper-mineralised region surrounding the primary osteons [28,29]. In contrast, in human bone, a thin mineralised region, the cementum line, surrounds the secondary osteons [30]. Both types of osteons have strong implications for fractures and are prominent sites for microcracking [26,31,32]. However, the yield strength of antler compact bone in the transverse direction is as low as 71 MPa [25], whereas the corresponding yield strength of human cortical bone is approximately 110–120 MPa.

The results showed that the present trial did not disturb the normal behaviour of the deer or antler regeneration. Therefore, antlers can be safely used as a novel method for implantation research with a high value in terms of the refinement of the animal trial for research purposes. Implants are easily inserted into the deer antler using a clinical procedure with traditional implantation instruments. Loading was successful and well controlled when added onto the abutments by self-developed loading devices. No implants were lost during the trial. Thus, the use of deer antlers combined with the novel loading device is a successful model for immediately loaded implant investigations.

Experimental specimens must be observed at different time intervals to understand the behaviour of the implants and surrounding tissues after immediate loading. However, animals must be sacrificed to perform a histological analysis of the bone bed. Moreover, in traditional animal models, the load application on implants is difficult to control because the installation of the loading device in the mouth or on top of other bone sites is affected by many factors such as food intake and physiological activity. In this study, a special self-developed loading device was placed externally at the antler and offered good accessibility and the application of a specific load without the abovementioned limiting factors. However, myiasis was prevented by a thorough velvet suture and application of a spray bandage at this external location, which would not have been a high risk inside the oral cavity. Using the loading device, the magnitude, direction and period of loading were controlled, thus avoiding other interfering factors and allowing the healing process to be monitored easily at different time intervals under immediate loading conditions.

After shedding the antler, the BMD of antler tissue from the unloaded specimens exhibited a similar value of 1.30 ± 0.11 g/cm^3^. These values were consistent with the results of the study by Chen *et al*. [25], who discovered that the total bone density of the antler was 1.35 ± 0.01 g/cm^3^. The antler density of the loaded specimens observed in this study was higher than that of the unloaded specimens in single animals. Thus, the density of the antler tissue around the implant continuously increases over the loading period. However, the density might be reduced after the complete osseointegration of the implant, as shown in previous studies [33,34].

The histological results revealed excellent osseointegration, with a mostly compact peri-implant antler bone exhibiting growth in the crestal direction along the implant surfaces. This finding is comparable to those of studies using other animal models [35–37]. Based on these findings, the deer antler is a novel and valid animal model that simulates the clinical implant healing process.

The deformation of the bone tissue by strain is inversely correlated to its stiffness. The higher the strain value, the lower the stiffness of the bone. The strain in the antler tissues of the loaded model was noticeably lower than that in the unloaded model. Thus, the osseointegration process of implants is faster under the loading condition than under the non-loading condition. These results are very important and present the deer antler as a novel, valid animal model to simulate the clinical implant healing process.

## Supporting information

S1 File The ARRIVE guidelines checklist. (DOCX)
