# Supplementary material for: Sika deer antler as a novel model to investigate dental implant healing: A pilot experimental study
Source: PLoS One. 2018 Jul 31;13(7):e0200957. doi: 10.1371/journal.pone.0200957 (PMC6067741; doi:10.1371/journal.pone.0200957)
Supplement: S1 File — (DOCX) [file pone.0200957.s001.docx]

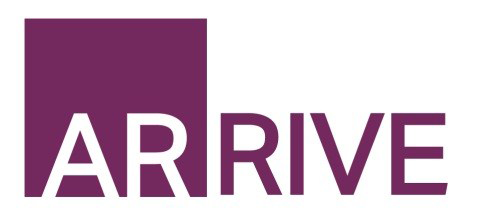


**The ARRIVE Guidelines Checklist**

**Animal Research: Reporting In Vivo Experiments**

Carol Kilkenny^1^, William J Browne^2^, Innes C Cuthill^3^, Michael Emerson^4^ and Douglas G Altman^5^

*^1^The National Centre for the Replacement, Refinement and Reduction of Animals in Research, London, UK, ^2^School of Veterinary Science, University of Bristol, Bristol, UK, ^3^School of Biological Sciences, University of Bristol, Bristol, UK, ^4^National Heart and Lung Institute, Imperial College London, UK, ^5^Centre for Statistics in Medicine, University of Oxford, Oxford, UK.*

|  | ITEM | RECOMMENDATION | Section/Paragraph |
| --- | --- | --- | --- |
| Title | 1 | Provide as accurate and concise a description of the content of the article as possible. | Sika deer antler as a novel model to investigate dental implant healing: A pilot experimental study |
| Abstract | 2 | Provide an accurate summary of the background, research objectives, including details of the species or strain of animal used, key methods, principal findings and conclusions of the study. | Dental implants are important tools for restoring the loss of teeth. The rapid growth and periodic regeneration of antlers make Sika deer a good and less invasive alternative model for studying bone remodelling in mammals. We developed a special loading device for antlers and analysed the bone reaction around unloaded implants and under immediate loading conditions until osseointegration occurred. In micro-computed tomography images, the density of antler tissue around the implants increased as the loading time increased. This finding was histologically confirmed by the good osseointegration observed in unloaded and loaded specimens. Antler tissue displays a similar healing process to human bone. The use of antler model is a promising alternative for implant studies that does not require animal sacrifice. |
| INTRODUCTION | | | |
| Background | 3 | a. Include sufficient scientific background (including relevant references to previous work) to understand the motivation and context for the study, and explain the experimental approach and rationale. | Dental implants are important tools for restoring the loss of single teeth and to fix tooth implant-supported fixed partial dentures after accidents, disease or age-related loss of teeth. Therefore, dental implants improve the quality of a patient’s life by improving aesthetics and phonetics and by decreasing the bone resorption processes in the alveolar ridge. In particular, immediately loaded dental implants offer fast dental restoration and pain relief. Research regarding complex bone remodelling processes occurring around dental implants is crucial to improve the function and acceptance of dental implants. Therefore, *in vivo* models are required, and animal trials have previously been conducted in pigs and dogs that were ultimately sacrificed. This limitation restricted an intensive investigation of bone tissue due to ethical reasons. |
|  |  | b. Explain how and why the animal species and model being used can address the scientific objectives and, where appropriate, the study’s relevance to human biology. | Deer antlers represent well-exposed and rapidly growing bones that change and regenerate periodically. Thus, antlers may be used as a good model for studying bone remodelling in mammals. Antlers start to grow from bony pedicles placed on the head of male Sika deer in winter or spring and are enveloped in vascularised velvet, the periosteum, during growth. The growth rate may reach up to 1.2 cm per day during the 70 days of the fastest growth period. In summer, growth ceases and antlers are completely mineralised; in addition, the velvet is shed, thereby exposing the bare bone of the so-called hard antlers from July to August. The development of an abscission layer across the base induces casting of the antlers to complete the cycle and to enable subsequent regrowth. |
| Objectives | 4 | Clearly describe the primary and any secondary objectives of the study, or specific hypotheses being tested. | Micro-computed tomography (µCT) and histological analyses were used to reveal changes in bone structure and density during healing and to understand the general ossification process and the specific reaction of bone around implants. Finite element analyses were used to observe the biomechanical properties of the implant and the surrounding antler tissue. |
| METHOD | | | |
| Ethical statement | 5 | Indicate the nature of the ethical review permissions, relevant licences (e.g. Animal [Scientific Procedures] Act 1986), and national or institutional guidelines for the care and use of animals, that cover the research. | All animals were handled according to the policies and principles established by the German Animal Welfare Act and approved by the North Rhine-Westphalia State Agency for Nature, Environment and Consumer Protection as the competent authority (Permission No.: LANUV NRW, 84-02.04.2014.A462). |
| Study design | 6 | For each experiment, give brief details of the study design including:  a. The number of experimental and control groups.  b. Any steps taken to minimise the effects of subjective bias when allocating animals to treatment (e.g. randomisation procedure) and when assessing results (e.g. if done, describe who was blinded and when).  c. The experimental unit (e.g. a single animal, group or cage of animals).  D. A time-line diagram or flow chart can be useful to illustrate how complex study designs were carried out. | Six healthy 4-year-old male captive bred and tamed Sika deer (Cervus nippon) were utilised in this study. The median weight of the animals was 64 kg (all 50 - 70 kg). There were two groups: immediately loaded implant group and unloaded implant group. Each group has 5 specimens, since the deer no. 6 was taken as control animal that did not receive an implant.  Animals were assigned randomly to different time points for implant removal, ranging from 3 to 6 weeks after surgery (deer no. 2: 3 weeks post-operation; deer no. 3: 4 weeks post-operation; deer no. 4: 5 weeks post-operation; and deer no. 5: 6 weeks post-operation). The same surgeon performed surgery, and histological and numerical evaluations were conducted by the same technician. They were blind to the treatment.  In the study, n refers to number of specimens.  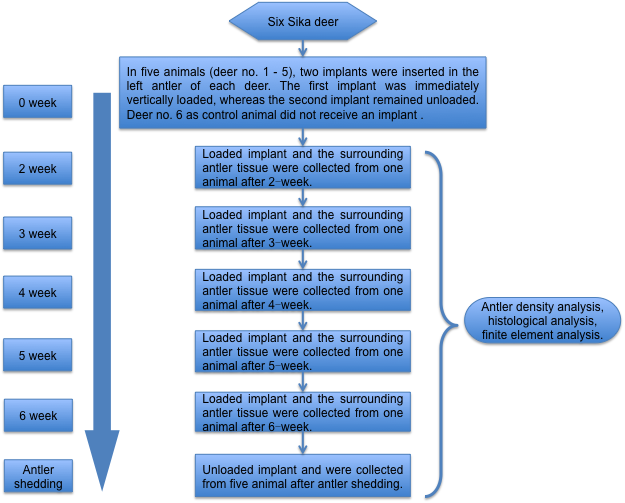 |
| Experimental procedures | 7 | For each experiment and each experimental group, including controls, provide precise details of all procedures carried out. For example:  a. How (e.g. drug formulation and dose, site and route of administration, anaesthesia and analgesia used [including monitoring], surgical procedure, method of euthanasia). Provide details of any specialist equipment used, including supplier(s).  b. When (e.g. time of day).  c. Where (e.g. home cage, laboratory, water maze).  d. Why (e.g. rationale for choice of specific anaesthetic, route of administration, drug dose used). | The animals were intramuscularly anaesthetised with 1.2 - 1.5 ml of Hellabrunn Mixture (100 mg of Ketamine and 125 mg of Xylazine per ml) via distance immobilisation using a carbon dioxide injection gun (DAN-INJECT JM Standard injection rifle, DAN-INJECT Smith GmbH, Walsrode, Germany). After placing the deer on a surgical table in right lateral recumbency, anaesthesia was monitored by a visual inspection of breathing, auscultation of the heart and lungs, continuous monitoring of the rectal temperature and pulse oximetry (LifeVet P, Eickemeyer Medizintechnik für Tierärzte KG, Tuttlingen, Germany). A continuous oxygen supply was ensured by the use of a nasal tube and a flow rate between 1 and 5 l/min, depending on the breathing and oxygen parameters. A continuous intravenous drip infusion was applied via the lateral saphenous vein. The non-steroidal, anti-inflammatory agent meloxicam (Meloxidyl 20 mg/ml ad us vet., Ceva Tiergesundheit, Düsseldorf, Germany) was injected intramuscularly. In addition, local analgesia was applied by injecting 3 - 5 ml of lidocaine beneath branches of the zygomatic nerve at the antler’s base (Lidocain 2 %, B. Braun Melsungen, Melsungen, Germany).  Before surgically raised the velvet from two 1.5 × 1.5-cm²-sized areas close to branches of the antler, disinfection was repeated with 70 % alcohol and a 1 % iodine tincture (Applichen GmbH, Darmstadt, Germany). The implants sites were prepared by sequential drilling with a Ø 2.2 mm and a Ø 2.8 mm cylindrical twist drill and 800 U/min under sterile saline irrigation according to the manufacturers protocol. In five animals (deer no. 1 - 5), two Straumann-Standard Plus Roxolid^®^ soft tissue level implants (Institut Straumann AG, Basel, Switzerland) with a length of 10 mm and a diameter of 3.3 mm were inserted in the left antler of each deer. The distance between two implants was 2.5 cm. The first implant received a ball abutment for transvelvet healing and the second received a closure screw. The tissues collected from the antlers of the sixth animal (deer no. 6, control animal that did not receive an implant) using a trepan drill were used as a control. The velvet was finally sutured using Serafit 4/0 (SERAG-WIESSNER GmbH & Co. KG, Naila, Germany) and subsequently covered by a Hansaplast spray bandage (Beiersdorf AG, Hamburg, Germany). The first implant was immediately vertically loaded via the custom-made loading device fixed with bone screws, whereas the second implant remained unloaded. The self-developed load control unit was attached to the right antler. No loading device was placed in the control animal.  All experiments were conducted in the light phase in July.  This study was conducted in Raptor Center and Wildlife Park Hellenthal and Endowed Chair of Oral Technology, University of Bonn.  The animals were intramuscularly anaesthetised with 1.2 - 1.5 ml of Hellabrunn Mixture via distance immobilisation using a carbon dioxide injection gun. In addition, local analgesia was applied by injecting 3 - 5 ml of 2 % lidocaine beneath branches of the zygomatic nerve at the antler’s base. The selected drug dose just follows the instruction of the drug and previous literatures. |
| Experimental animals | 8 | a. Provide details of the animals used, including species, strain, sex, developmental stage (e.g. mean or median age plus age range) and weight (e.g. mean or median weight plus weight range).  b. Provide further relevant information such as the source of animals, international strain nomenclature, genetic modification status (e.g. knock-out or transgenic), genotype, health/immune status, drug or test naïve, previous procedures, etc. | Six healthy 4-year-old male captive bred and tamed Sika deer (Cervus nippon). The median weight of the animals was 64 kg (all 50 - 70 kg). |
| Housing and husbandry | 9 | Provide details of:   1. Housing (type of facility e.g. specific pathogen free [SPF]; type of cage or housing; bedding material; number of cage companions; tank shape and material etc. for fish).   b. Husbandry conditions (e.g. breeding programme, light/dark cycle, temperature, quality of water etc for fish, type of food, access to food and water, environmental enrichment).  c. Welfare-related assessments and interventions that were carried out prior to, during, or after the experiment. | Sika deer are bred in captivity since centuries. They have been introduced in Europe in the 1880s and were kept by humans ever since out of different reasons (e. g. for hunting, for park maintenance, for zoological education, as hobby). Therefore, sika deer are used to human beings and keeping sika deer in captivity is not associated to animal welfare problems, behavioural disorders or stress in the animals. That is one of the reasons why sika deer are commonly kept and displayed by private owners, wildlife/game parks and zoological institutions. Husbandry requirements are known for this species and climatic conditions are convenient in Central Europe. Thus, there is no ethical dilemma in keeping sika deer in captivity prior and after the surgery.  During the surgery, a continuous oxygen supply was ensured by the use of a nasal tube and a flow rate between 1 and 5 l/min, depending on the breathing and oxygen parameters. A continuous intravenous drip infusion was applied via the lateral saphenous vein. The non-steroidal, anti-inflammatory agent meloxicam was injected intramuscularly. Post-operatively, the animals and wounds were inspected daily for the absence of wound healing complications, alterations in general health, abnormal behaviours and reduced food intake. The correct placement of the loading devices was monitored daily. |
| Sample size | 10 | 1. Specify the total number of animals used in each experiment, and the number of animals in each experimental group.   b. Explain how the number of animals was arrived at. Provide details of any sample size calculation used.   1. Indicate the number of independent replications of each experiment, if relevant. | Six deer were used in this study. Since two implants were inserted into the left antler and deer no. 6 was control animal without insertion of implant, there were 5 animals for loaded and unloaded implant group respectively. And the sample size was 5 for each goup.  This is a pilot study. |
| Allocating animals to experimental groups | 11 | 1. Give full details of how animals were allocated to experimental groups, including randomisation or matching if done.   b. Describe the order in which the animals in the different experimental groups were treated and assessed. | Per random number table, animals were assigned randomly to different time points for implant removal, ranging from 3 to 6 weeks after surgery (deer no. 2: 3 weeks post-operation; deer no. 3: 4 weeks post-operation; deer no. 4: 5 weeks post-operation; and deer no. 5: 6 weeks post-operation). |
| Experimental outcomes | 12 | Clearly define the primary and secondary experimental outcomes assessed (e.g. cell death, molecular markers, behavioural changes). | Bone mineral density, histological results, stresses and stain in antler tissue |
| Statistical methods | 13 | a. Provide details of the statistical methods used for each analysis.   1. Specify the unit of analysis for each dataset (e.g. single animal, group of animals, single neuron).   c. Describe any methods used to assess whether the data met the assumptions of the statistical approach. | No statistical methods were used. |
| RESULTS | | | |
| Baseline data | 14 | For each experimental group, report relevant characteristics and health status of animals (e.g. weight, microbiological status, and drug or test naïve) prior to treatment or testing. (This information can often be tabulated). | Signs of infections in the antler, behavioural changes and signs of impairment in the deer were not observed during the trial. The antlers of all animals were shed regularly during the subsequent spring and replaced by new and completely intact antlers within 2 - 3 months. |
| Numbers analysed | 15 | a. Report the number of animals in each group included in each analysis. Report absolute numbers (e.g. 10/20, not 50%2).  b. If any animals or data were not included in the analysis, explain why. | There were 4 loaded specimens and 3 unloaded specimens. For four immediately loaded time points (3, 4, 5, 6 weeks), each of them included one animal.  For two post-operative weeks loaded implant specimen, the thickness of antler tissue around implant was less than 3 mm. Therefore, this sample was not suitable for result analysis. Only three unloaded implant sample were collected after the antler shedding due to the fact that it was difficult to find all the shedded antlers in a spacious activity place for Sika deer. |
| Outcomes and estimation | 16 | Report the results for each analysis carried out, with a measure of precision (e.g. standard error or confidence interval). | The bone mineral density of antler tissue around the implant increased significantly during the healing period under immediate loading conditions. BMD values observed at 3, 4, 5, and 6 post-operative weeks were 0.31 ± 0.01 g/cm^3^, 0.92 ± 0.23 g/cm^3^, 1.54 ± 0.40 g/cm^3^, and 2.00 ± 0.53 g/cm^3^, respectively. For unloaded specimens, the BMD values for antler tissue were lower than those for loaded implants. For example, in the deer segment obtained 6 weeks after implant insertion, the BMD of antler tissue was 1.30 ± 0.11 g/cm^3^ around the unloaded implant and 2.00 ± 0.53 g/cm^3^ around the loaded implant.  Specimens carrying unloaded implants collected after antler shedding displayed signs of excellent osseointegration (Figs 3-4). Osseointegration appeared to be insufficient in the loaded specimens obtained at 3, 4 and 5 post-operative weeks. However, similar to the unloaded implant specimens, the loaded implant specimen retrieved at 6 post-operative weeks exhibited very good osseointegration and a compact peri-implant bone with good vascularisation (Figs 5-6).  Stresses in antler tissue increased from 2.4 MPa (3 weeks after surgery) to 6.5 MPa (5 weeks after surgery) after immediate loading and decreased to 1.7 MPa after 6 weeks of loading. The values in the unloaded models displayed a similar range (1.0 - 1.3 MPa). Strain in antler tissues decreased during the healing time for the loaded models, the 3-week (9,878 µstrain) and 6-week loaded models (49 µstrain), and illustrated the highest and lowest maximum strain values among the antler tissues (Fig 7). |
| Adverse events | 17 | 1. Give details of all important adverse events in each experimental group.   b. Describe any modifications to the experimental protocols made to reduce adverse events. | No adverse events. |
| DISCUSSION | | | |
| Interpretation/ scientific implications | 18 | 1. Interpret the results, taking into account the study objectives and hypotheses, current theory and other relevant studies in the literature.   b. Comment on the study limitations including any potential sources of bias, any limitations of the animal model, and the imprecision associated with the results.  c. Describe any implications of your experimental methods or findings for the replacement, refinement or reduction (the 3Rs) of the use of animals in research. | The results showed that the present trial did not disturb the normal behaviour of the deer or antler regeneration. Implants are easily inserted into the deer antler using a clinical procedure with traditional implantation instruments. Loading was successful and well controlled when added onto the abutments by self-developed loading devices. No implants were lost during the trial. Thus, the use of deer antlers combined with the novel loading device is a successful model for immediately loaded implant investigations. After shedding the antler, the BMD of antler tissue from the unloaded specimens exhibited a similar value of 1.30 ± 0.11 g/cm^3^. These values were consistent with the results of the study by Chen *et al*., who discovered that the total bone density of the antler was 1.35 ± 0.01 g/cm^3^. The antler density of the loaded specimens observed in this study was higher than the unloaded specimens in single animals. Thus, the density of the antler tissue around the implant continuously increases over the loading period. However, the density might be reduced after the complete osseointegration of the implant, as shown in previous studies. The histological results revealed excellent osseointegration, with a mostly compact peri-implant antler bone exhibiting growth in the crestal direction along the implant surfaces. FE results shown that the strain in the antler tissues of the loaded model was noticeably lower than in the unloaded model.  As a pilot study, the limited numbers of animal specimens. Besides, only two implant were inserted per antler since it was not possible to position more than one loading device per antler.  The results showed that the present trial did not disturb the normal behaviour of the deer or antler regeneration. The use of deer antlers combined with the novel loading device is a successful model for immediately loaded implant investigations that does not require animal sacrifice. |
| Generalisability/ translation | 19 | Comment on whether, and how, the findings of this study are likely to translate to other species or systems, including any relevance to human biology. | The histological results revealed excellent osseointegration, with a mostly compact peri-implant antler bone exhibiting growth in the crestal direction along the implant surfaces. This finding is comparable to studies using other animal models. |
| Funding | 20 | List all funding sources (including grant number) and the role of the funder(s) in the study. | This investigation was supported by Friadent GmbH (Mannheim, Germany) and Straumann GmbH (Freiburg, Germany) and the National Natural Science Foundation of China under Grant (11702231). |
